# Detection of *mcr-4* positive S*almonella enterica* serovar Typhimurium in clinical isolates of human origin, Italy, October to November 2016

**DOI:** 10.2807/1560-7917.ES.2018.23.2.17-00821

**Published:** 2018-01-11

**Authors:** Edoardo Carretto, Flavia Brovarone, Paola Nardini, Giuseppe Russello, Daniela Barbarini, Stefano Pongolini, Carlo Gagliotti, Alessandra Carattoli, Mario Sarti

**Affiliations:** 1 Clinical Microbiology Laboratory, IRCCS Arcispedale Santa Maria Nuova, Reggio Emilia, Italy; 2 Clinical Virology and Microbiology Laboratory - Fondazione IRCCS Policlinico San Matteo, Pavia, Italy; 3 Risk Analysis Unit, Istituto Zooprofilattico Sperimentale della Lombardia e dell'Emilia-Romagna, Sezione di Parma, Parma, Italy; 4 Regional Health and Social Agency of Emilia-Romagna, Bologna, Italy; 5 Department of Infectious Diseases, Istituto Superiore di Sanità, Rome, Italy; 6 Clinical Microbiology Laboratory, S. Agostino-Estense Hospital, Baggiovara, Italy

**Keywords:** *mcr-4*, *Salmonella enterica* serovar Typhimurium, colistin resistance, human, mobile colistin resistance, Italy

## Abstract

In this study we report the detection of the recently described *mcr-4* gene in two human isolates of *Salmonella enterica* serovar Typhimurium. The strains were isolated from faecal samples of two Italian patients with gastroenteritis, collected in 2016. The identified *mcr-4* genes (variant *mcr-4.2*) differed from the *mcr-4* gene originally described in a *Salmonella* strain of swine origin from Italy. *Salmonella* species could represent a hidden reservoir for *mcr* genes.

In the context of an analysis into the epidemiology of mobile colistin determinants, we investigated a collection of 106 human clinical isolates of Enterobacteriaceae. These isolates were obtained between January 2016 and October 2017 in two main hospitals in Emilia Romagna, Italy, namely IRCCS Arcispedale Santa Maria Nuova, Reggio Emilia and Sant’Agostino-Estense Hospital of Baggiovara. These isolates had originally been selected on the basis of their reduced susceptibility to colistin (MIC ≥ 2 mg/L). Among the 67 *Escherichia coli,* 27 *Klebsiella pneumoniae,* six *Salmonella* species and six other Enterobacteriaceae collected, we detected the *mcr-4* gene in two human isolates of *S. enterica* subsp. *enterica* monophasic variant of serovar Typhimurium. The *mcr-4* gene was not detected in any other isolate of the above collection; further analyses for other *mcr* genes are ongoing in these and additional isolates.

## Description of the isolates

The two *Salmonella* strains were isolated from faecal samples of outpatients with gastroenteritis who were referred to the Sant’Agostino-Estense Hospital. The first specimen, named AB-160, was collected in October 2016, the second one (AB-243) in November 2016. Patient data did not reveal any social, nosocomial or epidemiological links between the two patients. The disease was self-limiting and did not require hospitalisation or supportive therapy. According to European Committee on Antimicrobial Susceptibility Testing (EUCAST) breakpoints [1], the two isolates showed the same susceptibility profile on an automated instrument (Vitek-2, BioMérieux, France) and were resistant to ampicillin, piperacillin and colistin and susceptible to other beta-lactams.

After their isolation, the strains were serotyped by the Istituto Zooprofilattico Sperimentale della Lombardia e dell’Emilia-Romagna (IZSLER) following the White–Kauffmann–Le Minor scheme by slide agglutination with O and H antigen-specific sera (DID, Milan, Italy; Biogenetics, Padua, Italy). Both strains were identified as *S. enterica* subsp. *enterica* monophasic variant of serovar Typhimurium with antigenic formula 4,[5],12:i:- [2]. PFGE was performed according to the PulseNet protocol with *Xba*I digestion [3], and restriction profiles were analysed by BioNumerics 7.5 (Applied Maths, Saint-Martens – Latem, Belgium). The two isolates had different pulsotypes (AB-160: STYMXB.0083 and AB-243: STYMXB_PR.1681) but those were very similar, with a single band difference.

Colistin resistance was initially detected by using the automated instrument Vitek-2 (BioMérieux, France). They were confirmed in two different centres by using BMD-based commercial systems (SensiTest Colistin, Liofilchem, Italy, and Sensititre, ThermoFisher, US) and also with BMD performed in the IRCCS Arcispedale Santa Maria Nuova of Reggio Emilia according to the Clinical and Laboratory Standards Institute (CLSI) [4] and EUCAST (ISO standard method 20776-1) [5]. Three reference strains (*E. coli* ATCC 25922, *Pseudomonas aeruginosa* ATCC 27853 and *E. coli* NCTC 13846) were used as controls for susceptibility testing. Both *Salmonella* strains had a MIC for colistin of 8 mg/L by using SensiTest Colistin and the reference BMD, whereas Sensititre documented MICs of 4 mg/L and 8 mg/L for isolate AB-160 and AB-243, respectively.

## Detection of *mcr-4*

The DNA of the isolates was extracted using UltraClean microbial DNA isolation kit (MoBio Laboratories, US). The presence of *mcr* genes was investigated by different PCRs with previously described primers [6-10]. Both *Salmonella* isolates were positive for the *mcr-4* gene, using the internal primers Mcr-4 FW and Mcr-4 RV [7], and negative for the other four *mcr* genes. On these two isolates, the external primers Mcr-4 ext FW and Mcr-4 ext RV [7], targeting a fragment between 104 bases upstream of the start codon and 91 bases downstream of the stop codon of the *mcr-4* gene, gave no positive amplification. Furthermore, primers targeting the *repB* gene of the ColE10 plasmid did not give any amplification product [7].

To better characterise these *mcr-4* genes, we designed two additional primers derived from the sequence of the *mcr-4* gene published in GenBank, accession number NG_055659.1. The forward primer was Mcr4Fw_Out (AATGAGGTCAAGCTAGTAT) corresponding to bases 54–72 of NG_055659.1 (located at 47 to 29 bases upstream of the *mcr-4* start codon), and the reverse primer was Mcr4Rv_Out (ATATGTCACCCCTAAGATAA), matching bases 1,744–1,763 of NG_055659.1 (located at 18 to 37 bases downstream of the *mcr-4* stop codon). The annealing temperature was 54 °C.

An amplification product was obtained for both isolates and sequenced using both the above primers and previous internal primers. A sequence of 1,630 bp, identical for the two isolates, was obtained and deposited in GenBank with the accession number MG581979. MG581979 was a perfect match with the sequence NG_055659.1 across 1,629 bp, with a single nucleotide polymorphism (SNP) at position 1,001 of MG581979 (A to G), causing a transition of Q to R at amino acid position 331. This mutation defines this allele which we refer to as *mcr-4.2*.

## Discussion

Colistin is currently considered as the last-choice treatment for human infection caused by multidrug-resistant Gram-negative bacteria, despite well documented side effects and toxicity. Until recently, colistin resistance was extremely rare, mostly because of chromosomal mutations. In 2016, the first plasmid-mediated resistance to colistin was reported with the discovery of the *mcr-1* gene which encodes a phosphoethanolamine transferase [10]. Since then, different studies, based on retrospective analysis of strain collections, have demonstrated that this gene has a worldwide distribution [11], suggestive of a period of undetected dissemination. Currently, at least 12 different *mcr-1* gene variants have been acknowledged in GenBank (from mcr-1.1 to mcr-1.12: KP347127.1; KX236309.1; KY400027.1; KY041856.1; KY271416.1; KY352406.1; KY488488.1; KY685070.1; KY685071.1; MF176238.1; KY853650.1; LC337668.1). Furthermore, four additional *mcr* genes with varying homology to *mcr-1* have been discovered and named *mcr-2*, *mcr-3*, *mcr-4* and *mcr-5* [6-9]. The *mcr-4* gene was first described in 2017 in an Italian strain of *Salmonella enterica* subsp. enterica (monophasic variant of serovar Typhimurium 4,[5],12, i:-) of swine origin isolated in 2013 [7].

To our knowledge, this is the first report of *mcr-4*-positive bacterial isolates of human origin. The two *Salmonella* species belonged to the same serovar as the first *mcr-4*-positive *Salmonella* strain reported in a pig slaughtered in Italy [7]. That study had also reported 11 *mcr-4*-positive *E. coli* strains of animal origin in Spain and in Belgium, suggesting a dissemination of the novel gene among European countries and highlighting the need to evaluate possible risk and burden for human health [7]. The presence of the different *mcr-4.2* variant, the lack of amplification with external primers located outside of the coding sequence and the negative result for the ColE10 plasmid suggest that the genetic background of the *mcr-4* gene in our two human isolates is different from that in the *Salmonella* R3445 of animal origin [7]. Further analysis through whole genome sequencing is in progress to investigate the genetic homology of the two human isolates and their phylogenetic relatedness with the *Salmonella* isolate of veterinary origin.

This serovar of *Salmonella* is involved in most cases of human infection in the Emilia-Romagna region (personal communication: Stefano Pongolini, regional reference laboratory for surveillance of enteric pathogens at IZSLER, July 2017). Particularly in the period from July to August 2014, pulsotype STYMXB.0083 spread extensively thorough the provinces of Reggio Emilia and Modena (personal communication: Stefano Pongolini, IZSLER section of Parma, July 2017). On that occasion, the source of infection was identified as a pork product contaminated with the same *Salmonella* genotype as the human isolates. Since one of our strains had the pulsotype STYMXB.0083, further studies on the possible spread of mobile colistin resistance in a larger set of *Salmonella* strains, at least in our area, would be helpful to assess the risk to human health.

As demonstrated by the wide distribution of *mcr-1* worldwide [11], it could be hypothesised that also *mcr-4* has been spreading for many years undetected among *Enterobacteriaceae* in animals in European countries, with occasional transmission to humans. Rates of colistin resistance could be underestimated owing to the poor performance of different automated systems used routinely in clinical microbiology laboratories [12,13]. Epidemiology and distribution of *mcr* genes among animal and human isolates should be further investigated to evaluate their dissemination and possible transmission to strains which harbour other resistance determinants, such as extended-spectrum beta-lactamases and carbapenemases, as already demonstrated [12]. Acquisition of mobile colistin resistance by multidrug-resistant microorganisms in clinical settings would represent a serious problem for treatment and infection control [14]. *Salmonella* could represent a hidden reservoir for mobile colistin resistance determinants, considering the intensive use of colistin in animal farming that may have contributed to the selection of colistin-resistant strains [7]. Further studies and implementation of surveillance policies (improvement of laboratory methods for correct evaluation of phenotypical colistin resistance, e.g. by using BMD-based techniques as first-line antimicrobial susceptibility testing, and epidemiological analyses at local and national level, based on molecular detection of the different mobile resistance determinants, e.g. by multiplex PCRs) may be useful to understand the complexity and extent of the circulation of the different *mcr* genes and their impact in human health [15].
